# Supplementary material for: The Corticosterone–Glucocorticoid Receptor–AP1/CREB Axis Inhibits the Luteinizing Hormone Receptor Expression in Mouse Granulosa Cells
Source: Int J Mol Sci. 2022 Oct 18;23(20):12454. doi: 10.3390/ijms232012454 (PMC9604301; doi:10.3390/ijms232012454)
Supplement: Supplementary file 1 [file ijms-23-12454-s001.zip › Supplementary Data-S1.pdf]

pGL3-*Lhcgr*-promoter sequence

The red font highlights represent mutant sequences

WT:

ATATGAATTCCAACCTTTCTCCTACAGAGGAAGAAACTAAAGGCCAAGCCACAATGCTGAACACAGATCA
GAAGAACCTCCTGGTGTCTCCATTTCCCAGTGCTTCTGCTATTGGAGTGCACAGTGAGACAGCTCTTCCT
AGAATAACCCACTGTTCTCAGAGGGTCAGTGGTGGCTGTAAATATACACGGGGTAGTGAGGAGTAGGTAT
ATTCTCTAGGACACACACACACATAGATATATAGGAGATATGATATGGTATGATATGATATGATATAAAA
TAAAATAAAAAGTAAAGGCTACCCATAAAACATGAGCCTTGAAATAAATGCCATTCAGCCACTTTTATTC
TCATTTATTCTCTCTGCCTTAATGGCTCCATGGTAGAGAAGTCAGAAAGCTTTCACCGGCACTACCAAAA
CCTGACAATGCTAAATGAGGAGGAGACACAGCATAAACCCGGGATACATCTGGGCCATCGCTCAGTTCCT
ATGTAGATACATCCAGGATGGCTCGGGACAAACCAGATAAAGCTGAGACGCTCGTCTCTTGTGACTCGGA
GTATCTGCTCTAGTCTCAAGCCCAGATTTCACTGTTCATTTAATCGTTCATCAGATATGTGCGGAGCGAC
ACCTACGAACCAGAAGCCGTGACGAGCCACTCAGTACTAAGGTTAACTGAAAGGGGAAAGCAAAATCACG
TCCTTCAGTGCTTTTTAAGCTACAAGCTTTAGAACCAGGGCCTATTAAGCTATTCTGAGTTAGAGAAGGC
TCCCATGGAAGGGGCAGCAGTCTGTCTTGAAATACAACCAGAAGCTTGCCCAGTGGAATACCAGAAAAGG
AAGACGGCATCAAGCAAGCTTTGGGAAGTGTGCACAAAGACATGAAAATGTAAATCAGCAAAGATCAAAT
CCCACACAATGTGAGCAGAGCTGGGTGATTTCAAAACAAGAGCAATAGAATAATGATCATCTACCTTACA
GGTTTGGGCAAAAGACTGCAAGCTG**GTGACTGA**GGAAGCTGCCTTTCAGAACAAGAACTCTGCCCTGCTT
TCTGTCTTGAAAGCTTTAGATGTCTTTACAGGGCTCAACATCCACCTCTTCCAGAAAAAAGAAAATTGCT
AATATTTCTAGAGTCCAAAATCAATCCTTGGAAGGAACGATGCCTAATGAACACCGTAGAGGGGAAATGA
CCTGTTCCTGGGGCTGCTGTAAGATCAGGTTTTCTTTCTGCGGGGTTTAGAATCACAGACCCTATAGCAG
AGGTTTTCCACTAGTAAGAGACAGGTGGGTGACAGACAGGAGTCAAAAAGCACTCCTCCATCCACAAGTT
GGGTTACCATAGAAACTGTATACCTTGTTGGGTATTATGTTCCTGCTACAAATGCCCTAAGAGTCTGGTG
TAACAGACATGATGTGCGGCAAGGCATCTATCACAGTCTTAAAGCACGGTGCACACCCGTTGAGGCCATT
TTACCCACTCACATAGCAAGCCTTTGTTAACTAAAAAAAAAGAATACAAAGTGAGTCCAAACTGAATTTT
GCCCTCAAGGGCTTCACAGTGCAGTGGGGATATATAGGACAGACCCAAATAGCTATGCTGAGGAGAACAG
TTACAACCAAGAGAAGCAGAGAGATCAGTGAGGAAGCAGAGAATGGAGAGATTTCTTGTCAAGAATGGAG
GAGCAGAGCCTAGAGCTAAGACACAGCAGGCCTGGCACTTTGGCTCTTCCGATGTGGATATGGACAGAGA
CATCTCATAGAACCAAGACAAGTCTCATGGGGACCATGGCAGAGCAGAGTTCAAAGCCTCACTCTAAGCG
CTTACCACACATGACTCTATATTCTAACCCTCAGTTCATTTGCAAGCTTAGAGCAGAGGGCAAGTCTTAG
GGAAACAGCAATGGGGTGGCTGGCCACAGTCCCAGGTCAAGGAGAACAGGGACAGGCGGTGAGAGGGGAG
GGCTGGAGCGGGCGGGGGCCGGCGGGTGGGAAGGCAGGCC

M1, mutated Creb binding site :

ATATGAATTCCAACCTTTCTCCTACAGAGGAAGAAACTAAAGGCCAAGCCACAATGCTGAACACAGATCA
GAAGAACCTCCTGGTGTCTCCATTTCCCAGTGCTTCTGCTATTGGAGTGCACAGTGAGACAGCTCTTCCT
AGAATAACCCACTGTTCTCAGAGGGTCAGTGGTGGCTGTAAATATACACGGGGTAGTGAGGAGTAGGTAT
ATTCTCTAGGACACACACACACATAGATATATAGGAGATATGATATGGTATGATATGATATGATATAAAA
TAAAATAAAAAGTAAAGGCTACCCATAAAACATGAGCCTTGAAATAAATGCCATTCAGCCACTTTTATTC

TCATTTATTCTCTCTGCCTTAATGGCTCCATGGTAGAGAAGTCAGAAAGCTTTCACCGGCACTACCAAAA
CCTGACAATGCTAAATGAGGAGGAGACACAGCATAAACCCGGGATACATCTGGGCCATCGCTCAGTTCCT
ATGTAGATACATCCAGGATGGCTCGGGACAAACCAGATAAAGCTGAGACGCTCGTCTCTTGTGACTCGGA
GTATCTGCTCTAGTCTCAAGCCCAGATTTCACTGTTCATTTAATCGTTCATCAGATATGTGCGGAGCGAC
ACCTACGAACCAGAAGCCGTGACGAGCCACTCAGTACTAAGGTTAACTGAAAGGGGAAAGCAAAATCACG
TCCTTCAGTGCTTTTTAAGCTACAAGCTTTAGAACCAGGGCCTATTAAGCTATTCTGAGTTAGAGAAGGC
TCCCATGGAAGGGGCAGCAGTCTGTCTTGAAATACAACCAGAAGCTTGCCCAGTGGAATACCAGAAAAGG
AAGACGGCATCAAGCAAGCTTTGGGAAGTGTGCACAAAGACATGAAAATGTAAATCAGCAAAGATCAAAT
CCCACACAATGTGAGCAGAGCTGGGTGATTTCAAAACAAGAGCAATAGAATAATGATCATCTACCTTACA
GGTTTGGGCAAAAGACTGCAAGCTG**ACTGACAC**GGAAGCTGCCTTTCAGAACAAGAACTCTGCCCTGCTT
TCTGTCTTGAAAGCTTTAGATGTCTTTACAGGGCTCAACATCCACCTCTTCCAGAAAAAAGAAAATTGCT
AATATTTCTAGAGTCCAAAATCAATCCTTGGAAGGAACGATGCCTAATGAACACCGTAGAGGGGAAATGA
CCTGTTCCTGGGGCTGCTGTAAGATCAGGTTTTCTTTCTGCGGGGTTTAGAATCACAGACCCTATAGCAG
AGGTTTTCCACTAGTAAGAGACAGGTGGGTGACAGACAGGAGTCAAAAAGCACTCCTCCATCCACAAGTT
GGGTTACCATAGAAACTGTATACCTTGTTGGGTATTATGTTCCTGCTACAAATGCCCTAAGAGTCTGGTG
TAACAGACATGATGTGCGGCAAGGCATCTATCACAGTCTTAAAGCACGGTGCACACCCGTTGAGGCCATT
TTACCCACTCACATAGCAAGCCTTTGTTAACTAAAAAAAAAGAATACAAAGTGAGTCCAAACTGAATTTT
GCCCTCAAGGGCTTCACAGTGCAGTGGGGATATATAGGACAGACCCAAATAGCTATGCTGAGGAGAACAG
TTACAACCAAGAGAAGCAGAGAGATCAGTGAGGAAGCAGAGAATGGAGAGATTTCTTGTCAAGAATGGAG
GAGCAGAGCCTAGAGCTAAGACACAGCAGGCCTGGCACTTTGGCTCTTCCGATGTGGATATGGACAGAGA
CATCTCATAGAACCAAGACAAGTCTCATGGGGACCATGGCAGAGCAGAGTTCAAAGCCTCACTCTAAGCG
CTTACCACACATGACTCTATATTCTAACCCTCAGTTCATTTGCAAGCTTAGAGCAGAGGGCAAGTCTTAG
GGAAACAGCAATGGGGTGGCTGGCCACAGTCCCAGGTCAAGGAGAACAGGGACAGGCGGTGAGAGGGGAG
GGCTGGAGCGGGCGGGGGCCGGCGGGTGGGAAGGCAGGCC

M2, mutated Creb binding site :

ATATGAATTCCAACCTTTCTCCTACAGAGGAAGAAACTAAAGGCCAAGCCACAATGCTGAACACAGATCA
GAAGAACCTCCTGGTGTCTCCATTTCCCAGTGCTTCTGCTATTGGAGTGCACAGTGAGACAGCTCTTCCT
AGAATAACCCACTGTTCTCAGAGGGTCAGTGGTGGCTGTAAATATACACGGGGTAGTGAGGAGTAGGTAT
ATTCTCTAGGACACACACACACATAGATATATAGGAGATATGATATGGTATGATATGATATGATATAAAA
TAAAATAAAAAGTAAAGGCTACCCATAAAACATGAGCCTTGAAATAAATGCCATTCAGCCACTTTTATTC
TCATTTATTCTCTCTGCCTTAATGGCTCCATGGTAGAGAAGTCAGAAAGCTTTCACCGGCACTACCAAAA
CCTGACAATGCTAAATGAGGAGGAGACACAGCATAAACCCGGGATACATCTGGGCCATCGCTCAGTTCCT
ATGTAGATACATCCAGGATGGCTCGGGACAAACCAGATAAAGCTGAGACGCTCGTCTCTTGTGACTCGGA
GTATCTGCTCTAGTCTCAAGCCCAGATTTCACTGTTCATTTAATCGTTCATCAGATATGTGCGGAGCGAC
ACCTACGAACCAGAAGCCGTGACGAGCCACTCAGTACTAAGGTTAACTGAAAGGGGAAAGCAAAATCACG
TCCTTCAGTGCTTTTTAAGCTACAAGCTTTAGAACCAGGGCCTATTAAGCTATTCTGAGTTAGAGAAGGC
TCCCATGGAAGGGGCAGCAGTCTGTCTTGAAATACAACCAGAAGCTTGCCCAGTGGAATACCAGAAAAGG
AAGACGGCATCAAGCAAGCTTTGGGAAGTGTGCACAAAGACATGAAAATGTAAATCAGCAAAGATCAAAT
CCCACACAATGTGAGCAGAGCTGGGTGATTTCAAAACAAGAGCAATAGAATAATGATCATCTACCTTACA
GGTTTGGGCAAAAGACTGCAAGCTG**GTGACTGA**GGAAGCTGCCTTTCAGAACAAGAACTCTGCCCTGCTT
TCTGTCTTGAAAGCTTTAGATGTCTTTACAGGGCTCAACATCCACCTCTTCCAGAAAAAAGAAAATTGCT

AATATTTCTAGAGTCCAAAATCAATCCTTGGAAGGAACGATGCCTAATGAACACCGTAGAGGGGAAATGA
CCTGTTCCTGGGGCTGCTGTAAGATCAGGTTTTCTTTCTGCGGGGTTTAGAATCACAGACCCTATAGCAG
AGGTTTTCCACTAGTAAGAGACAGGTGGGTGACAGACAGGAGTCAAAAAGCACTCCTCCATCCACAAGTT
GGGTTACCATAGAAACTGTATACCTTGTTGGGTATTATGTTCCTGCTACAAATGCCCTAAGAGTCTGGTG
TAACAGACATGATGTGCGGCAAGGCATCTATCACAGTCTTAAAGCACGGTGCACACCCGTTGAGGCCATT
TTACCCACTCACATAGCAAGCCTTTGTTAACTAAAAAAAAAAGAATACAAA**GTGAGTCC**AAACTGAATTTT
GCCCTCAAGGGCTTCACAGTGCAGTGGGGATATATAGGACAGACCCAAATAGCTATGCTGAGGAGAACAG
TTACAACCAAGAGAAGCAGAGAGATCAGTGAGGAAGCAGAGAATGGAGAGATTTCTTGTCAAGAATGGAG
GAGCAGAGCCTAGAGCTAAGACACAGCAGGCCTGGCACTTTGGCTCTTCCGATGTGGATATGGACAGAGA
CATCTCATAGAACCAAGACAAGTCTCATGGGGACCATGGCAGAGCAGAGTTCAAAGCCTCACTCTAAGCG
CTTACCACACATGACTCTATATTCTAACCCTCAGTTCATTTGCAAGCTTAGAGCAGAGGGCAAGTCTTAG
GGAAACAGCAATGGGGTGGCTGGCCACAGTCCCAGGTCAAGGAGAACAGGGACAGGCGGTGAGAGGGGAG
GGCTGGAGCGGGCGGGGGCCGGCGGGTGGGAAGGCAGGCC
