# Supplementary material for: The Corticosterone–Glucocorticoid Receptor–AP1/CREB Axis Inhibits the Luteinizing Hormone Receptor Expression in Mouse Granulosa Cells
Source: Int J Mol Sci. 2022 Oct 18;23(20):12454. doi: 10.3390/ijms232012454 (PMC9604301; doi:10.3390/ijms232012454)

Figure 1A

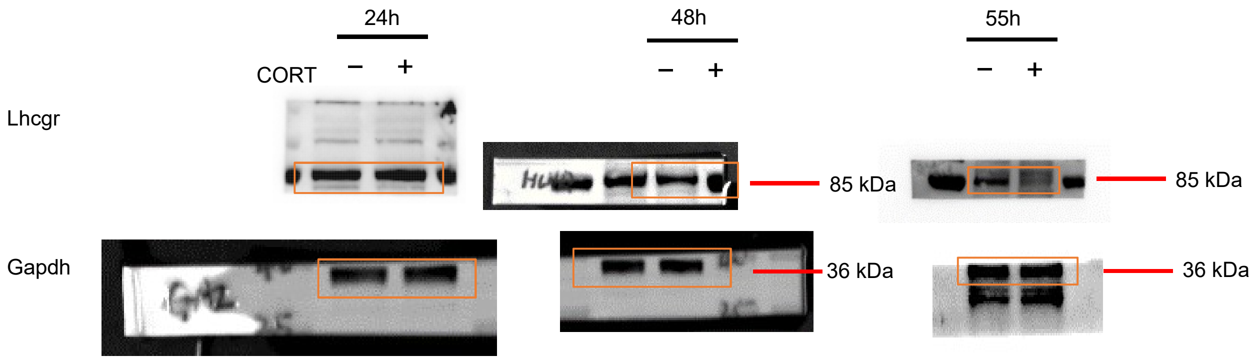

Figure 1E

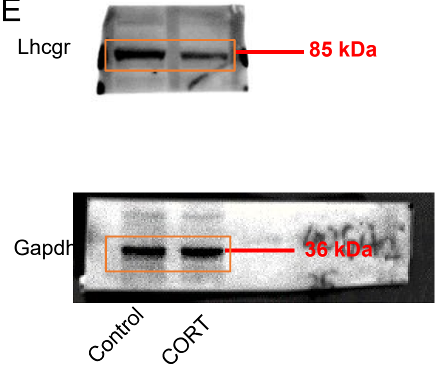

Figure 2B

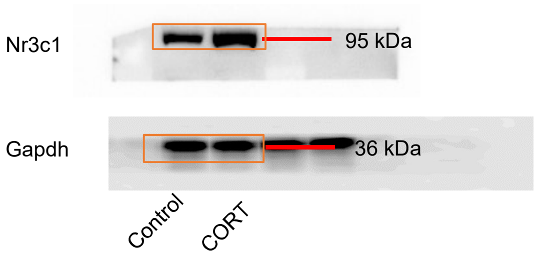

Figure 2D

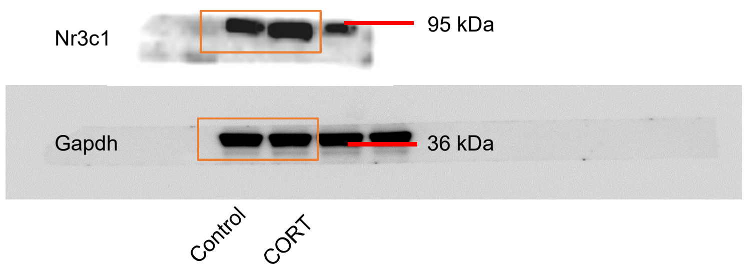

Figure 3A

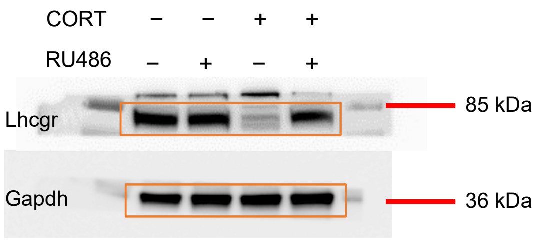

Figure 3D

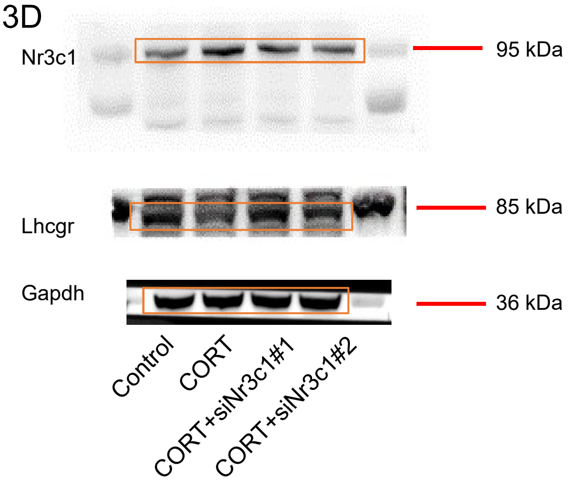

Figure 4A

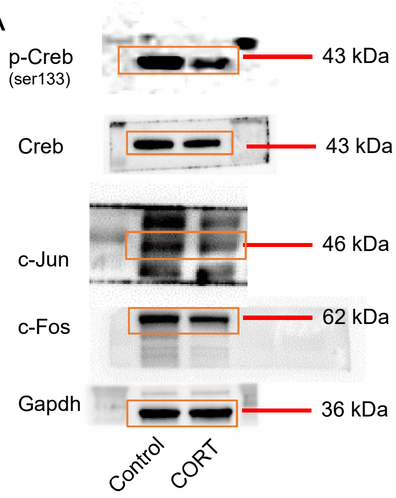

Figure 5A

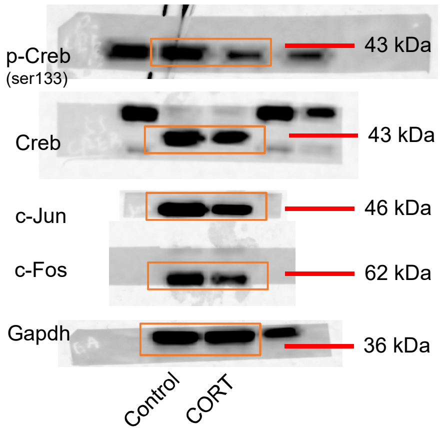

Figure 5D

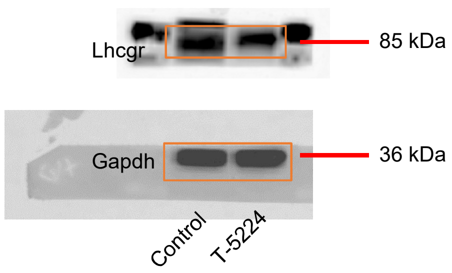

Figure 5E

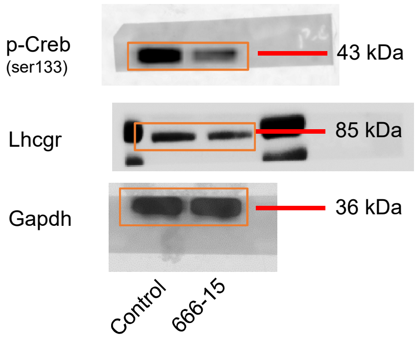

Figure 6A

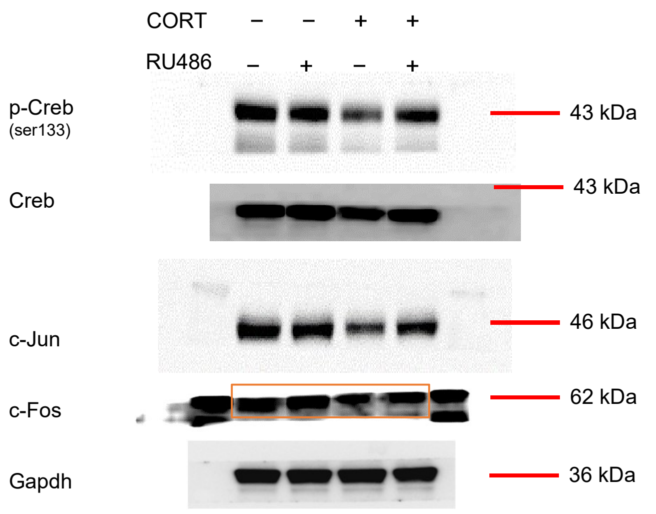

Figure 6F

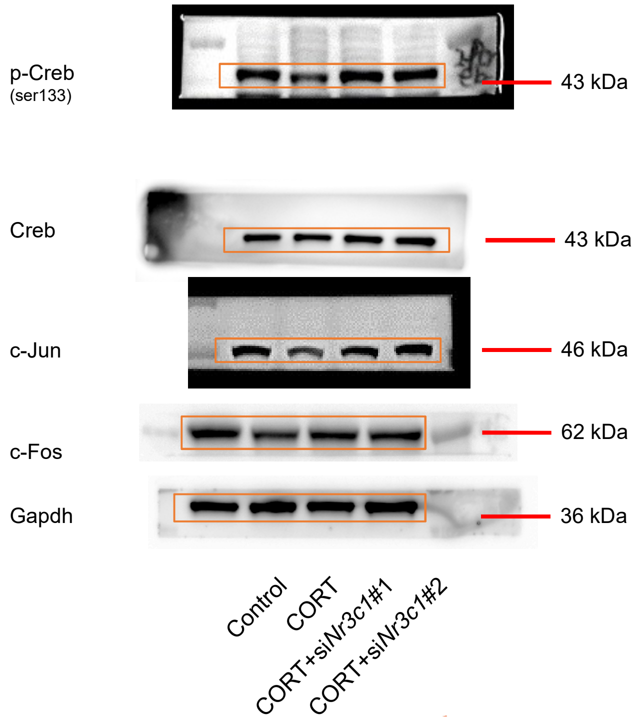

Supplement: Supplementary file 1 [file ijms-23-12454-s001.zip › Supplementary Data-S2.pdf]
