# Supplementary material for: The Corticosterone–Glucocorticoid Receptor–AP1/CREB Axis Inhibits the Luteinizing Hormone Receptor Expression in Mouse Granulosa Cells
Source: Int J Mol Sci. 2022 Oct 18;23(20):12454. doi: 10.3390/ijms232012454 (PMC9604301; doi:10.3390/ijms232012454)
Supplement: Supplementary file 1 [file ijms-23-12454-s001.zip › Supplementary Tables S1 and S2.pdf]

Table S1. Primers for qRT-PCR

Gene Name	Primer Sequence (5'–3')	GenBank Accession NO.
Lhcgr	*F: GCTGACCTACCCTAGCCACT*<br>*R: CAGAGTGATGAAGCGTCTCGT*	*NM_001364898.1*
Creb1	*F: TGTACCACCGGTATCCATGC*<br>*R: TGGATAACGCCATGGACCTG*	*NM_133828.2*
Jun	*F: GGCACATCACCACTACACCG*<br>*R: GTTCTGGCTATGCAGTTCAGC*	*NM_010591.2*
Fos	*F: GTGAAGACCGTGTCAGGAGG*<br>*R: GATCTGTCTCCGCTTGGAGT*	*NM_010234.3*
Nr3c1	*F: AGTCAAGGTTTCTGCGT*<br>*R: CCATCACTTTTGTTTCG*	*NM_008173.4*
Gapdh	*F: AGGCGCTGAAAAAGGGTGAT*<br>*R: GGTCCAGGGTTTCTTACTCCT*	*NM_001289726.1*

Table S2. siRNA sequences

siRNA Name	Sense（5'-3'）	Antisense（5'-3'）
si*Nr3c1*#1	*GGCAGUGUGAAAUUGUAUATT*	*UAUACAAUUUCACACUGCCTT*
si*Nr3c1*#2	*GGUGUUAUAUGCAGGAUAUTT*	*AUAUCCUGCAUAUAACACCTT*
Scrambled siRNA	*UUCUCCGAACGUGUCACGUTT*	*ACGUGACACGUUCGGAGAATT*
